# Hybridization of papain molecules and DNA-wrapped single-walled carbon nanotubes evaluated by atomic force microscopy in fluids

**DOI:** 10.1038/s41598-023-31927-8

**Published:** 2023-03-24

**Authors:** Masaki Kitamura, Kazuo Umemura

**Affiliations:** Department of Physics, Tokyo University of Science, 1-3 Kagurazaka, Shinjuku, 1628601 Japan

**Keywords:** Applications of AFM, Biosensors

## Abstract

Although various conjugates of single-walled carbon nanotubes (SWNTs) and biomolecules, such as nanobiosensors and nanobiodevices, have been reported, the conjugation of papain and SWNTs have not been reported because of the formation of unexpected aggregates. In this study, atomic force microscopy (AFM) in liquid was used to investigate the interactions between papain and DNA-wrapped SWNTs (DNA–SWNTs) at two different pH values (pH 3.0 and 10.5). The direct AFM observation of the mixture of papain and DNA–SWNTs confirmed the aggregation of papain molecules with DNA–SWNTs in the buffer solutions. The numerous and non-uniform adsorption of papain molecules onto DNA–SWNTs was more pronounced at pH 3.0 than that at pH 10.5. Furthermore, thick conjugates appeared when papain and DNA–SWNTs were simultaneously mixed. The near-infrared photoluminescence spectra of the SWNTs drastically changed when the papain molecules were injected into the DNA–SWNT suspension at pH 3.0. Thus, the regulation of electrostatic interactions is a key aspect in preparing optimal conjugates of papain and DNA–SWNTs. Furthermore, although previous papers reported AFM images of dried samples, this study demonstrates the potential of AFM in liquid in evaluating individual bioconjugates of SWNTs.

## Introduction

Atomic force microscopy (AFM) is a unique tool for observing biomolecules and bioconjugates in aqueous solutions^1–4^. As most biomolecules deform when they are dried, their native structures or bioconjugates are difficult to observe by AFM. Thus, AFM observation in fluids has been recognized as an attractive approach for analyzing native structures and molecular interactions. For example, the structures of DNA molecules in dried and wet forms were compared by AFM in air and liquids^5–7^. The diameters of DNA molecules dramatically decrease when DNA is dried on a mica surface^8–10^. Moreover, because proteins are usually utilized in liquid, investigations in liquid have advanced understanding of their native behavior and potential applications^11–14^. Radmacher et al. directly observed the enzyme reactions in liquids using AFM^12^. These approaches have been further developed using high-speed AFM systems^15–19^.

An effective approach for observing bioconjugates is using AFM in liquids. Thus, the conjugation of DNA and DNA-binding proteins has been intensively studied by AFM in fluids^2,20^, thereby visualizing the binding sites of the proteins. Conjugates of biomolecules and nanomaterials, such as single-walled carbon nanotubes (SWNTs), which have been developed for biological and medical applications are attractive targets for AFM in liquids. Although previous studies have mostly observed DNA-wrapped SWNTs (DNA–SWNTs) in the air, Hayashida et al. observed DNA–SWNT hybrids in aqueous solutions^21^. The diameters of the same DNA–SWNT hybrids drastically changed according to the environment and forces between the hybrids and the AFM probe. Assuming a constant SWNT diameter, the fluctuations in the diameters of the hybrids can be ascribed to the plasticity of DNA molecules on SWNT surfaces.

SWNTs exhibit extraordinary mechanical, electrical, and optical properties^22–24^. In particular, the near-infrared (NIR) photoluminescence (PL) of SWNTs can be applied to detect biological reactions on SWNT surfaces^25–28^. As SWNTs exhibit NIR PL when irradiated with visible light, the NIR PL fluctuates in intensity and emission wavelengths with the environmental changes on the SWNT surfaces^29–33^. For example, when oxidants or reductants, such as hydrogen peroxide and vitamin C, are injected into DNA–SWNT suspensions, the PL intensity of DNA–SWNTs drastically decreases and increases with the oxidant and reductant content, respectively^29,30^. When certain molecules are adsorbed on SWNT surfaces, the PL spectra are affected by the adsorbed molecules^31–33^. Using the unique optical responses of SWNTs, the fabrication of bioconjugates with SWNTs has been proposed for nanobiosensors and nanobiodevices^34–41^. Similar approaches to utilizing the electrical abilities of SWNTs have also been reported^42,43^. For example, conjugates of glucose oxidase and SWNTs have been proposed as nanosized glucose sensors^44,45^.

In this study, we investigated the interactions of papain molecules with DNA–SWNTs using AFM in liquids. Papain is a cysteine protease with a high thermostability^46–48^. Although several enzymes lose their activity at 60 °C, papain exhibits sufficient enzyme activity at this temperature. Thus, various biological and medical applications have been proposed considering its thermostability^49,50^. Therefore, the conjugation of papain molecules and DNA-SWNT hybrids can be used as a biosensor for papain enzymes to sense proteins at high temperatures. In addition, papain molecules on SWNT surfaces can become an alternative substrate used to monitor enzyme activation reactions using the change of PL of SWNTs. However, even with the in-depth report on the fabrication of various types of biomolecules and SWNTs hybrids, the fabrication of conjugates of papain molecules and SWNTs is yet to be reported. In particular, attaching papain molecules to DNA–SWNT hybrids can form aggregates of the papain/DNA–SWNT conjugates that can be visibly recognized, thereby resulting in the absence of papain–SWNT hybrids. In addition, the formation of aggregates is an interesting phenomenon leading to speculation that the aggregations were caused by the high pI of papain enzymes of 8.6^51^ because of the water solubility of DNA–SWNTs and papain. Therefore, it is necessary to investigate the conjugations at two pH values across the pI of papain enzymes.

In our experiments, mixtures of papain molecules and DNA–SWNT hybrids were observed to evaluate the adsorption of papain molecules on DNA–SWNT hybrids. Although the fabrication of bioconjugates has been widely reported^1–4^, the microscopic evaluation of the adsorption of biomolecules onto SWNT surfaces using AFM in liquids is yet to be reported. Thus, our approach is valuable for understanding the adsorption mechanism of biomolecules onto SWNTs, and providing useful information for establishing optimal procedures for the fabrication of bioconjugates with SWNTs.

## Results and discussion

**Figure 1:**
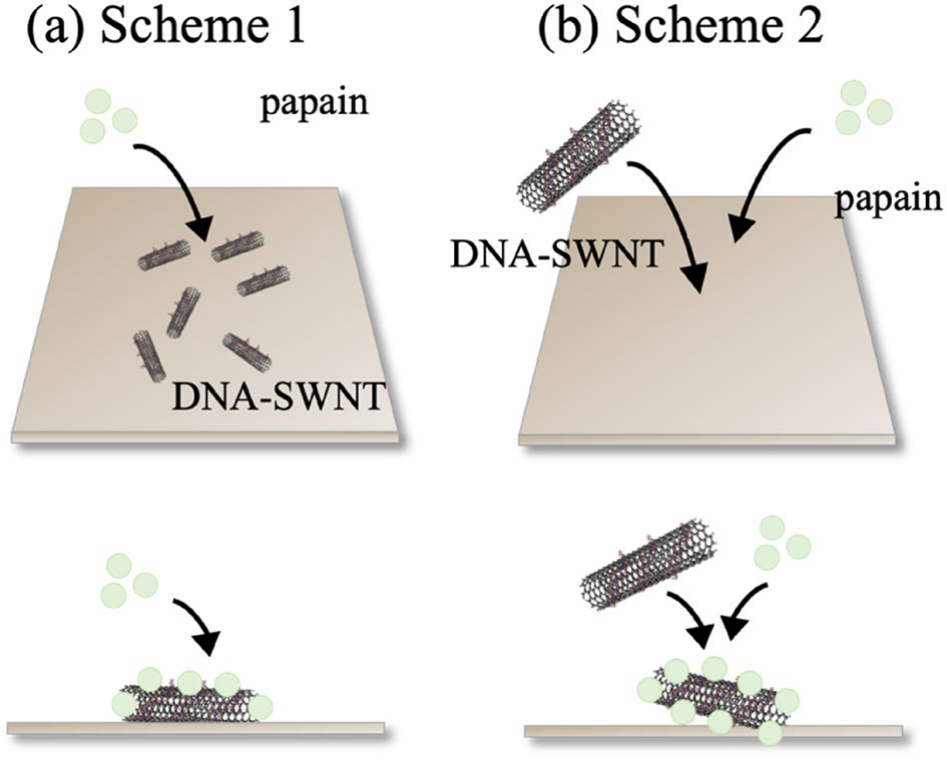
Observation of DNA–SWNTs and papain on the AP-treated mica substrate. (**a**) Scheme 2: DNA–SWNT solution was dropped first, followed by the injection of the papain solution. (**b**) Papain and DNA–SWNT solutions were successively dropped. All schemes were conducted at pH 3.0 and 10.5.

As mentioned, when we simply mixed DNA-SWNT hybrids and papain molecules in a tube the formation of aggregates was visually recognizable (Supplementary Fig. S1). We speculated that the aggregations were caused by pH conditions, and thus relatively high pH levels were tested to clearly observe changes so that the mechanism of the aggregations would be easily understood. Figure 1 shows the scheme used in our study of AFM in fluids. In Scheme 1, DNA–SWNT hybrids were first attached to a 3-aminopropyltriethoxysilane (AP)–mica surface set in an AFM liquid cell. The sample was observed once with a 2.0 mL buffer solution. Two types of buffer solutions, namely 10 mM citric acid (pH 3.0) and 10 mM boric acid (pH 10.5), were used to evaluate the effects of pH. Papain molecules were injected into the AFM liquid cell, and the sample was observed again in the buffer solutions. In Scheme 2, the DNA–SWNT suspension was dropped onto the AP–mica surface, and the papain solution was immediately added. In this scheme, the papain and DNA–SWNT hybrids are expected to have sufficient degrees of freedom.

**Figure 2:**
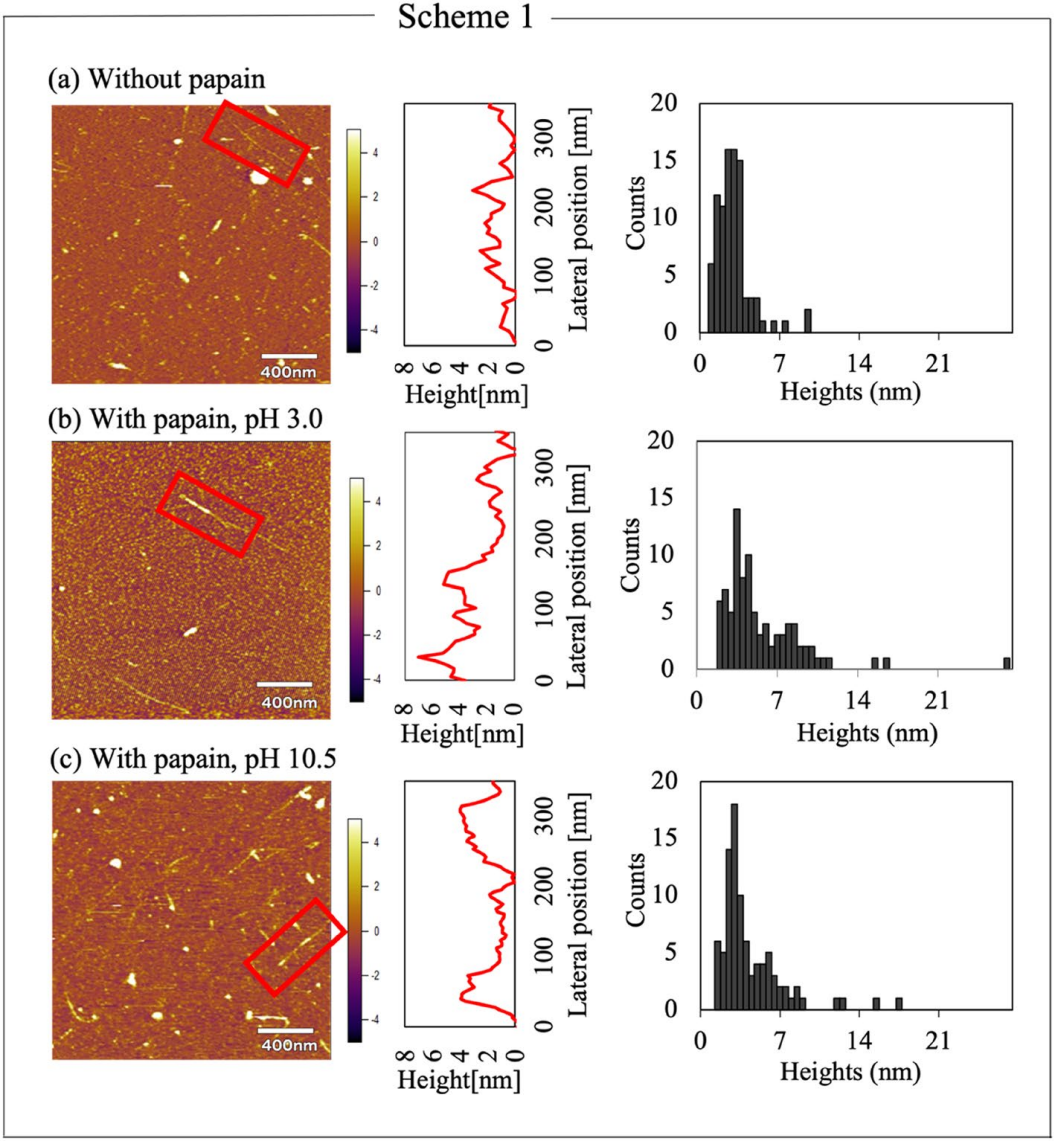
AFM images and height histograms of the DNA–SWNTs (**a**) before the injection of papain solution, (**b**) after the injection under pH 3.0, and (**c**) after the injection of papain under pH 10.5. (**a**) Was analyzed in an AFM-liquid condition after dropping the DNA–SWNT solution onto the AP-treated mica substrates. (**b**) and (**c**) Were analyzed similarly after injection of the papain solution. The histogram was illustrated using the height data corrected with those of gold colloids.

Figure 2 shows the results of the Scheme 1 experiments (Supplementary Fig. S2 shows magnified results). DNA–SWNT hybrids were clearly observed in 10 mM citric acid buffer solution (pH 3.0 (Fig. 2a). Subsequently, as 10 μL DNA–SWNT suspension was dropped onto an AP-mica surface and the AFM liquid cell was filled with 2.0 mL buffer solution, similar images were obtained using 10 mM boric acid buffer solution at pH 10.5 (Supplementary Fig. S3). To reobserve the samples, 50 μL papain solution was injected. The observation was carried out immediately after stabilizing the perturbation caused by the injection. Figure 2b shows a typical AFM image of the mixture of papain and DNA–SWNTs in the citric acid buffer solution (pH 3.0). The diameter of the DNA–SWNT hybrids partially increased under pH 3.0 (square in Fig. 2b). Moreover, the morphologies differed from those in Fig. 2a, suggesting the partial adsorption of papain molecules on the DNA–SWNT hybrids. The cross-sections of the observed DNA–SWNT hybrids in the squares are indicated on the right side of each image. For DNA–SWNTs without papain, the heights were approximately less than 2 nm. Meanwhile, the height of the partial area that papain molecules might adsorbed on was almost 6 nm, whereas that of the other areas was less than 2 nm.

Similar experiments were carried out at pH 10.5 with boric acid buffer solution. The diameter of DNA–SWNTs partially increased; however, the number of DNA–SWNTs onto which papain molecules were adsorbed was lower than that at pH 3.0. Although the height of SWNTs was affected by differences in chirality, the significantly higher areas than for those without papain molecules were observed in the cross sections. This implied that papain molecules were adsorbed onto DNA-SWNT hybrids at pH 10.5 as well. For the numerical analysis, 90 cross-sections from randomly selected 30 hybrids were summarized as histograms since SWNTs are not homogeneously flat. Because the average height of 100 gold colloids was 3.57 ± 0.76 nm (manufacturer height is 5.00 nm), all heights for height analysis were calibrated with this ratio. The average heights and standard deviations of DNA–SWNTs with and without papain molecules were 2.70 ± 1.51 and 5.40 ± 3.67 nm at pH 3.0, and 2.61 ± 1.44 and 4.10 ± 2.83 nm at pH 10.5, respectively. The median heights were 2.49, 4.16, 2.43, and 3.14 nm, respectively (Supplementary Fig. S4). *T*-tests from these results revealed that the height of DNA–SWNTs increased after the injection of papain solution under both pH conditions (pH 3.0: *N* = 90, *t* (89) = $-$ 6.44, p = $1.00\times {10}^{-8}<0.05$, pH 10.5: *N* = 90, *t* (89) = $-$ 6.12, *p* = $1.00\times {10}^{-8}<0.05$). Thus, papain molecules could be adsorbed by DNA–SWNTs at pH 10.5 and 3.0. However, from the histograms, the average height of DNA–SWNTs at pH 3.0 was slightly higher than that at pH 10.5 (*N* = 90, *t* (89) = $-$ 2.71, *p* = 8.04 $\times {10}^{-3}<0.05$). This implies that more papain molecules could attach to DNA–SWNTs at pH 3.0 than at pH 10.5, and hence interactions were more favorable. Further, the isoelectric point (PI) of papain should be considered. The PI of the papain molecules was approximately 8.75, suggesting the negative charge at pH 10.5.

For the control experiments, papain molecules and DNA–SWNT hybrids on AP–mica surfaces in fluids were observed by AFM (Supplementary Fig. S3). Globular and rod structures were observed for papain and DNA–SWNTs, respectively. The size of the observed papain molecules was approximately 3–4 nm. When the concentration of papain solutions was dense enough to cover all surfaces of the AP-mica (1.0 mg/mL) the papain molecules were adsorbed on the AP–mica surfaces at pH 3.0 and 10.5. However, since the surfaces were positively charged, a greater amount of papain molecules was observed at pH 10.5 than 3.0 at the low concentration (0.05 mg/mL) (Supplementary Fig. S3b). Some of the papain molecules were adsorbed on the DNA–SWNT surfaces under the Scheme 1 experiments. This suggests the attractive interaction between papain and DNA–SWNT hybrids, which compete with those between papain and AP–mica.

In addition, the reverse procedure in Scheme 1 was examined for comparison. In Supplementary Fig. S5, papain molecules were first deposited on an AP–mica surface, followed by the injection of the DNA–SWNT suspension into the AFM liquid cell. In this scheme, the AP–mica surface is occupied by papain molecules, as shown in Supplementary Fig. S5(a). As a result, several aggregates were observed at pH 3.0. As shown by Supplementary Fig. S5(b), although numerous samples were observed with several tips, the images were influenced by tip artifacts, which implies the tip might have lifted up the debris from the mica surface. Since the tip artifacts were not confirmed in scheme 1, the conjugations might be formed by several DNA-SWNT hybrids instead since the hybrids were immobilized on the mica surface in scheme 1. Therefore, the debris might have ejected from the conjugation. However, it was confirmed that papain molecules tend to adsorb onto the DNA–SWNTs at pH 3.0, whereas these aggregates were not observed at pH 10.5.

**Figure 3:**
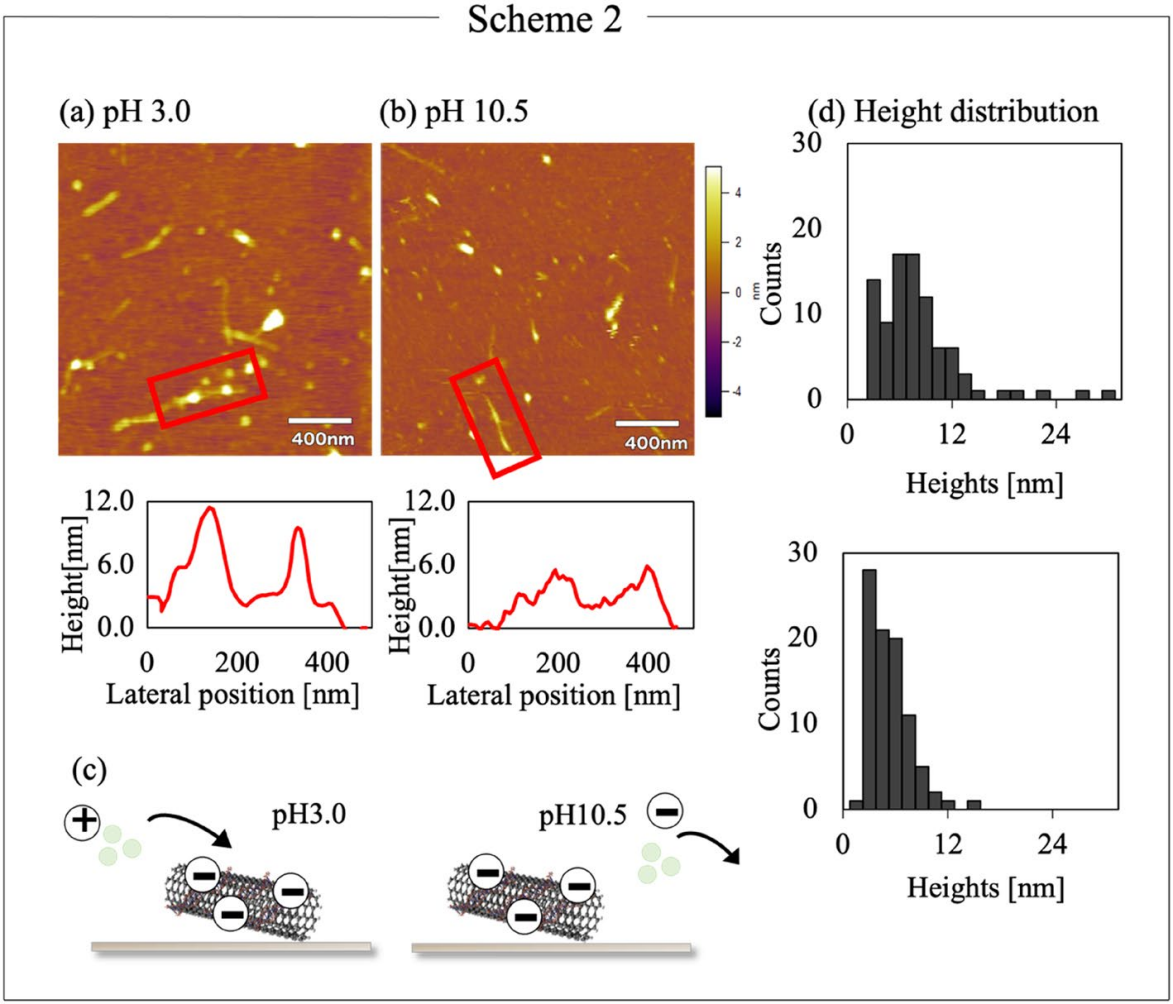
AFM images and height histograms of the DNA–SWNTs mixed with the papain at (**a**) pH 3.0 and (**b**) pH 10.5. The experiments were analyzed in AFM liquid condition after successively dropping the DNA–SWNT and papain solutions onto the AP-treated mica substrates. (**c**) Interactions of the papain molecules and DNA–SWNTs at pH 3.0 and 10.5. (**d**) The histograms illustrate the height data corrected with those of the gold colloids.

Figure 3 shows the results of the Scheme 2 experiment (Supplementary Fig. S6 shows magnified results). Figure 3a shows the typical AFM image of a mixture of papain and DNA–SWNT hybrids in the citric acid buffer solution at pH 3.0. Onto the AP-mica surface, 10 μL DNA–SWNT suspension was dropped, immediately followed by the dropping of 10 μL papain solution (1 mg/mL). After 10 min of incubation at 25 °C, 2.0 mL citric acid buffer solution (pH 3.0) was added for the AFM observation. The red markers in Fig. 3a indicate that the DNA–SWNT hybrid was decorated with papain molecules. Six DNA–SWNTs heterogeneously covered with papain molecules were observed in the image. In addition, the observed DNA-SWNT hybrids appear blurry, indicating the tip might have picked up some debris from the surface of the mica for the same reason. Supplemental Fig. 6(a) confirms the existence of the DNA-SWNT hybrids (red markers) together with the debris (blue markers). Figure 6(a) also shows that papain molecules attached to DNA-SWNT hybrids at their thicker parts. Figure 3b shows the typical AFM image obtained by similar experiments at pH 10.5 with the boric acid buffer solution. Some DNA–SWNT hybrids were covered with papain molecules. The red markers indicate one DNA–SWNT hybrid with papain molecules. Similarly, thin DNA–SWNT hybrids were observed at pH 10.5. From the cross-sections of the DNA–SWNTs (red markers), parts with larger heights were clearly observed on the conjugates. These parts at pH 3.0 have a larger height than those at pH 10.5. A flat surface of DNA–SWNTs is obtained with the homogeneous attachment of the papain molecules on the surface of DNA–SWNTs. Figure 3c shows the expected interaction between the papain molecules and DNA–SWNTs in citric acid (Scheme 2, pH 3.0) and boric acid (pH 10.5) buffer solutions. Considering the PI of the papain enzyme and DNA of 8.6 and 2.0, respectively, the papain molecules were positively charged at pH 3.0 and negatively charged at pH 10.5, whereas the DNA particles were negatively charged at pH 3.0 and 10.5. This suggests that the papain molecule and DNA–SWNT has an attractive interaction at pH 3.0 and a repulsive interaction at pH 10.5, as shown in Fig. 3c.

Figure 3d shows the height histograms of 90 parts (three randomly selected parts of each of the 30 DNA–SWNTs) in Scheme 2. The average heights with a standard deviation of the DNA–SWNTs at pH 3.0 and 10.5 were 7.30 ± 4.81 and 4.60 ± 2.83 nm, respectively (Supplementary Fig. S7). The median height of the DNA–SWNTs at pH 3.0 and 10.5 were 6.27 and 4.26 nm, respectively (*N* = 90,* t* (89) = 5.02, *p* = 2.60 $\times {10}^{-6}<0.05$). The numerical analysis supports the electrostatic interactions as major factors in determining the binding of papain molecules to the DNA–SWNT hybrids. For a more in-depth discussion, the histograms in Figs. 2b and 3a, which were obtained at pH 3.0, were compared. The DNA–SWNT surfaces tend to adsorb the papain molecules. The average height in Fig. 2b (5.40 ± 3.67 nm) was smaller than that in Fig. 3a (7.30 ± 4.81 nm), which can be ascribed to the different deposition procedures (*N* = 90, *t* (89) = 2.84, *p* = 5.66 $\times {10}^{-3}<0.05$). In particular, Fig. 2 shows the results from first attaching the DNA–SWNT hybrids, followed by injecting the papain solution, whereas Fig. 3 denotes the results of immediately mixing the DNA–SWNT suspension and papain solution on the AP–mica surface.

**Figure 4:**
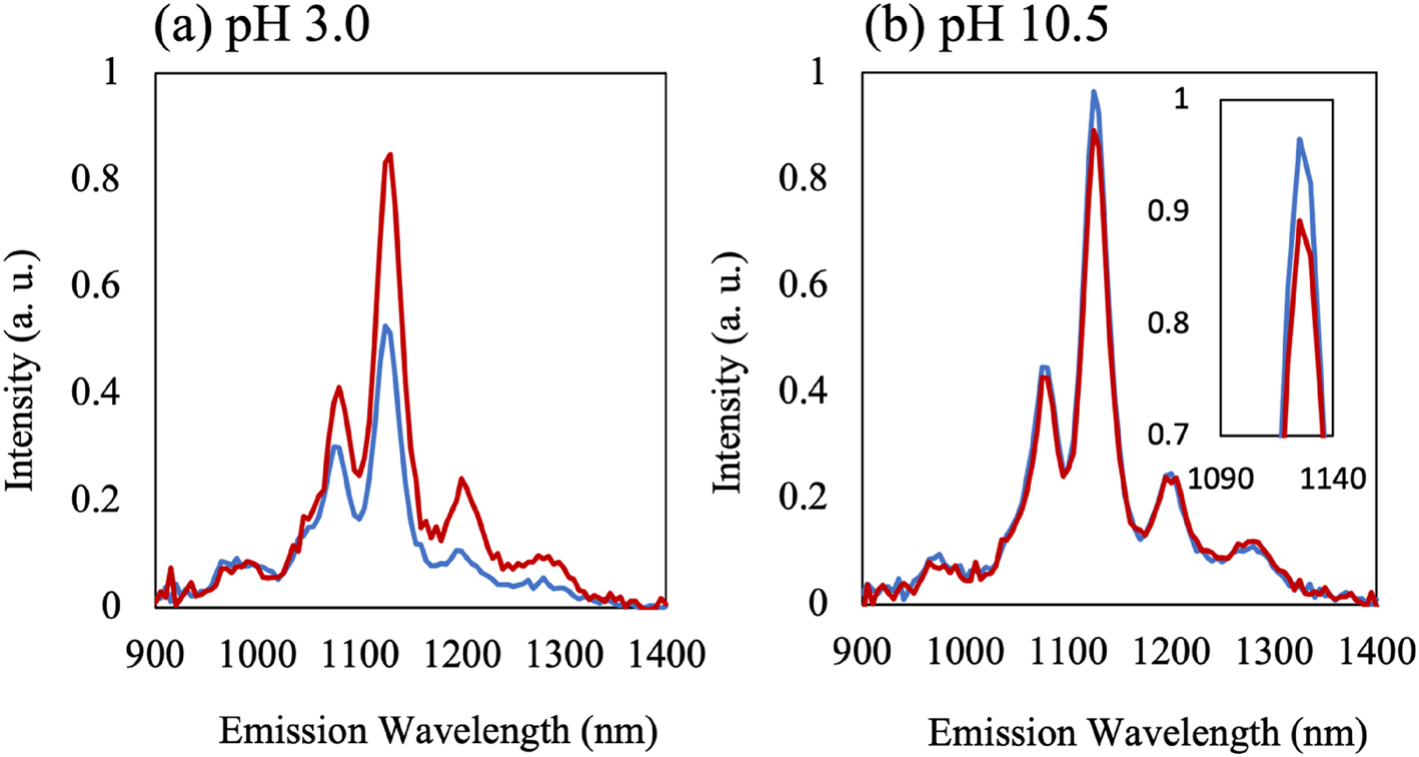
NIR-PL Spectra at (**a**) pH 3.0 and (**b**) 10.5. The blue and red spectra refer to that before and after the injection of the papain solution, respectively.

To confirm the effects of pH on the interaction between the papain molecules and DNA–SWNTs, PL measurements were performed on a mixture of DNA–SWNT hybrids and papain molecules. As SWNTs exhibit NIR PL when irradiated with visible light, the adsorption of papain molecules onto the DNA–SWNT surfaces drastically changes the PL spectra, as detected by measuring the NIR PL. In addition, as aggregates are observed after mixing the papain solution with the DNA–SWNT solution, the samples were stirred before the measurements, and the PL spectra were immediately measured. Figure 4a and b show the PL spectra of the mixtures of the DNA–SWNT hybrid and papain solutions at pH 3.0 and 10.5, respectively. For the measurements, 24 μL DNA–SWNT solution was diluted with 1176 μL buffer solutions in a cuvette (final SWNT concentration of 9.8 μg/mL), and the PL spectra were measured once. Furthermore, 24 μL papain solution was injected into the cuvette, and the PL was measured after mixing. Assuming from our results that DNA–SWNTs and papain are rods (500 nm in length, 1 nm in diameter) and spheres (3 nm in diameter), respectively, the molar ratio of SWNTs: papain was estimated to be approximately 1:30. For the PL measurements, the excitation wavelength was 730 nm, and the measured PL range was 900–1200 nm.

Several PL peaks originate from the chiralities of SWNTs. Focusing on the peak at 1125 nm, which originates from the (9, 4) chirality of SWNTs, the PL intensity of the peak increased by 67.1 ± 8.45% after adding the papain solution at pH 3.0, whereas it slightly decreased by 8.24 ± 1.07% after the addition of the papain solution at pH 10.5. Although further research is necessary to understand the mechanism, the addition of papain clearly imposed contradicting results on the change in the PL spectra under two pH conditions. This indicates the PL spectra changed at pH 3.0 while barely changing at pH 10.5 when considering the change in volume caused by the addition of papain solution. Our results from AFM experiments verified the greater adsorption of papain molecules at pH 3.0. Thus, the adsorption of papain molecules onto DNA–SWNT hybrids tended to increase the PL intensity. As pH is controlled by the buffer solutions, fluctuations in the pH values before and after injection of the papain molecules were considered negligible. Thus, the results further suggest the potential of the DNA–SWNT hybrids to detect enzymatic reactions of the papain molecules.

## Conclusion

In this study, a mixture of papain molecules and DNA–SWNT hybrids was observed using AFM in liquids. The papain molecules were not uniformly adsorbed on the DNA–SWNT surfaces, especially at pH 3.0. The electrostatic interactions between the papain molecules and DNA–SWNT hybrids are considered important parameters for establishing nanobiodevices of papain and DNA–SWNT hybrids. Furthermore, the PL spectra of the DNA–SWNTs effectively detected the adsorption of papain molecules at pH 3.0. This suggests a potential application for detecting enzymatic reactions using DNA–SWNT hybrids.

## Materials and methods

### Materials

SWNTs produced by a high-pressure carbon monoxide process method (HiPco) were purchased from HS27-122, Raymor Industries Inc. (QC, Canada). Double-stranded DNA (deoxyribonucleic acid sodium salt from salmon testes, No. D1626-250 MG) and papain (164-00172) were purchased from Sigma-Aldrich Co. LLC (MO, USA). All the chemicals were used as received.

### Preparation of DNA–SWNTs

First, DNA was dissolved in 10 mM tris(hydroxymethyl)-aminomethane (Tris–HCl buffer pH 8.0) to prepare a 1.0 mg/mL DNA solution. For each dispersion, the solution was placed in a bath-type ultrasonicator (80 W) for 180 min at 0 °C, which was maintained on ice. The solution was gently shaken in an ice bath for 180 min. To prepare 0.5 mg/mL DNA–SWNT solution, 0.55 mg SWNTs were dissolved in 1.10 mL prepared DNA solution. The solution was well-dispersed using a probe-type sonicator (VCX130, Sonics & Materials, Inc., CT, USA) for 90 min at a power of 2 W and temperature of 0 °C, which was controlled by ice. The solution was centrifuged for 180 min at 15,000 rpm and 8 °C. Finally, 70% supernatant was collected and stored in a refrigerator at 5 °C until further use. Papain solutions (1.0 mg/mL) were prepared by dissolving papain in 10 mM citric acid (pH 3.0) and 10 mM boric acid (pH 10.5) buffer solutions, respectively. The solutions were stored in a refrigerator at 0 °C, which was controlled by ice, until further use.

### AFM analysis

AFM observations were performed in AC-AFM mode (MFP-3D microscope, Asylum Research, CA, USA) in the liquid state and were used for the silicon cantilever PPP-NCSTR-W (NANOSENSORS, Nanoworld Neuchatel, Switzerland). All processes were conducted under pH 3.0 (citric acid buffer solution) and 10.5 (boric acid buffer solution), respectively.

Three types of samples were prepared for the AFM analysis. The first sample was prepared as follows: 10 μL prepared DNA–SWNT solution was deposited onto muscovite mica pretreated with AP. Subsequently, the mica surface was washed using 1000 μL buffer solution after incubation for 10 min. Mica was attached to the bottom of a closed fluid cell (CFC, 939.010, Asylum Research, CA, USA) and soaked in 2000 μL buffer solution. AFM measurements were conducted in the fluid at room temperature using the CFC after incubation for 15 min. From the CFC, 1000 μL buffer solution was removed, and a mixture of 50 μL prepared papain solution and 950 μL buffer solution were injected into the container. AFM measurements were conducted in the fluid after incubation for 15 min at room temperature.

The second sample was prepared as follows: 10 μL prepared DNA–SWNT solution was deposited onto the mica, which was immediately followed by the deposition of 10 μL papain solution onto the mica. Mica was attached to the bottom of the CFC and soaked in 2000 μL buffer solution. AFM measurements were conducted in the fluid using the CFC after incubation for 15 min at room temperature.

Finally, we deposited 10 μL prepared papain solution onto muscovite mica pretreated with AP. Subsequently, the mica surface was washed using 1000 μL buffer solution after incubation for 10 min. Mica was attached to the bottom of a closed fluid cell (CFC, 939.010, Asylum Research, CA, USA) and soaked in 2000 μL buffer solution. AFM measurements were conducted in fluid at room temperature using the CFC after incubation for 15 min. From the CFC, 1000 μL buffer solution was removed, and a mixture of 10 μL prepared DNA–SWNT solution and 990 μL buffer solution was injected into the container. AFM measurements were conducted in the fluid after incubation for 15 min at room temperature.

All experiments were conducted in triplicate. For the structural analysis, 30 DNA–SWNT hybrids of a size of more than 250 nm were randomly chosen. The heights of 90 points from the 30 hybrids were collected. Then *t*-tests were conducted to determine significant differences. For the calibration of our AFM, 5 nm gold colloids (EM.GC5, BBI Solutions, UK; mean diameter of 4.6–6.0 nm; coefficient of variation:$\le$ 15%) was diluted 100 times with water. Subsequently, 10 μL solution was dropped onto the mica for the AFM observations in the air. Then 100 gold colloids were randomly chosen, and the average height was determined to compare the height given by the manufacturer and attain the calibration ratio for the actual height of DNA-SWNT hybrids. In this study, all the DNA-SWNT hybrids for the height analysis were calibrated with the ratio.

### PL spectroscopy

PL spectroscopy measures the emission wavelengths in the NIR region. In this study, PL measurements were performed using the prepared DNA–SWNT and papain solutions. The excitation and emission wavelength ranges were 730 and 900–1400 nm, respectively. All measurements were conducted under pH 3.0 and 10.5, respectively. For the NIR-PL spectroscopy, 1176 μL buffer solution and 24 μL prepared DNA–SWNT solution were mixed in a cuvette, and the spectra were recorded initially. Subsequently, 24 μL papain solution was added to the cuvette, which was incubated with stirring for 10 min. After incubation, the PL of the samples was measured again. The experiments were conducted in triplicate.

## Supplementary Information

Supplementary Figures.

## Data Availability

All date generated or analyzed during this study are included in this published article [and its supplementary information files].

## References

[1] Lillehei PT, Bottomley LA (2000). Scanning probe microscopy. Anal. Chem..

[2] Matsumoto T, Mikamo-Satoh E, Takagi A, Kawai T (2012). Single molecular observation of DNA and DNA complexes by atomic force microscopy. Curr. Pharm. Biotechnol..

[3] Niemeyer CM (2001). Nanoparticles, proteins, and nucleic acids: Biotechnology meets materials science. Angew. Chem. Int. Ed..

[4] Walter, N. G. *Single Molecule Tools, Part B: Super-Resolution, Particle Tracking, Multiparameter, and Force Based Methods* (Academic Press, 2010).

[5] Bustamante C, Keller D (1995). scanning force microscopy. Biol. Phys. Today.

[6] Hansma HG, Hoh JH (1994). Biomolecular imaging with the atomic force microscope. Annu. Rev. Biophys. Biomol. Struct..

[7] Kominami H, Kobayashi K, Yamada H (2019). Molecular-scale visualization and surface charge density measurement of Z-DNA in aqueous solution. Sci. Rep..

[8] Hansma HG, Bezanilla M, Zenhausern F, Adrian M, Sinsheimer RL (1993). Atomic force microscopy of DNA in aqueous solutions. Nucleic Acids Res..

[9] Liu Z (2005). Imaging DNA molecules on mica surface by atomic force microscopy in air and in liquid. Microsc. Res. Tech..

[10] Vesenka J, Manne S, Yang G, Bustamante C, Henderson E (1993). Humidity effects on atomic force microscopy of gold-labeled DNA on mica. Scan. Microsc..

[11] Fritz M (1995). Imaging globular and filamentous proteins in physiological buffer solutions with tapping mode atomic force microscopy. Langmuir.

[12] Radmacher M, Fritz M, Hansma HG, Hansma PK (1994). Direct observation of enzyme activity with the atomic force microscope. Science.

[13] Zhuravel R (2016). Atomic force microscopy characterization of kinase-mediated phosphorylation of a peptide monolayer. Sci. Rep..

[14] Stühn L, Auernhammer J, Dietz C (2019). pH-depended protein shell dis-and reassembly of ferritin nanoparticles revealed by atomic force microscopy. Sci. Rep..

[15] Ando T (2005). High-speed atomic force microscopy for capturing dynamic behavior of protein molecules at work. e-J. Surface Sci. Nanotechnol..

[16] Colom A, Casuso I, Rico F, Scheuring S (2013). A hybrid high-speed atomic force–optical microscope for visualizing single membrane proteins on eukaryotic cells. Nat. Commun..

[17] Preiner J (2015). High-speed AFM images of thermal motion provide stiffness map of interfacial membrane protein moieties. Nano Lett..

[18] Suzuki Y (2013). High-speed atomic force microscopy combined with inverted optical microscopy for studying cellular events. Sci. Rep..

[19] Shibata M, Uchihashi T, Ando T, Yasuda R (2015). Long-tip high-speed atomic force microscopy for nanometer-scale imaging in live cells. Sci. Rep..

[20] Bennink ML, Nikova DN, van der Werf KO, Greve J (2003). Dynamic imaging of single DNA–protein interactions using atomic force microscopy. Anal. Chim. Acta.

[21] Hayashida T, Umemura K (2016). Atomic force microscopy of DNA-wrapped single-walled carbon nanotubes in aqueous solution. Colloids Surf. B.

[22] Gong H, Peng R, Liu Z (2013). Carbon nanotubes for biomedical imaging: The recent advances. Adv. Drug Deliv. Rev..

[23] Münzer AM, Michael ZP, Star A (2013). Carbon nanotubes for the label-free detection of biomarkers. ACS Nano.

[24] Yan Y (2015). Carbon nanotube catalysts: Recent advances in synthesis, characterization and applications. Chem. Soc. Rev..

[25] Gaufres E (2009). Enhancement of semiconducting single-wall carbon-nanotube photoluminescence. Opt. Lett..

[26] Lee AJ (2011). Bright fluorescence from individual single-walled carbon nanotubes. Nano Lett..

[27] Nakamura G, Narimatsu K, Niidome Y, Nakashima N (2007). Green tea solution individually solubilizes single-walled carbon nanotubes. Chem. Lett..

[28] Tanaka Y, Niidome Y, Nakashima N (2009). In situ photoluminescence spectroelectrochemistry of single-walled carbon nanotubes with nine different chiral indices. Chem. Lett..

[29] Ishibashi Y, Ito M, Homma Y, Umemura K (2018). Monitoring the antioxidant effects of catechin using single-walled carbon nanotubes: Comparative analysis by near-infrared absorption and near-infrared photoluminescence. Colloids Surf. B.

[30] Umemura K, Ishibashi Y, Ito M, Homma Y (2019). Quantitative detection of the disappearance of the antioxidant ability of catechin by near-infrared absorption and near-infrared photoluminescence spectra of single-walled carbon nanotubes. ACS Omega.

[31] Matsukawa Y, Ohura S, Umemura K (2020). Effect on near-infrared absorption spectra of DNA/single-walled carbon nanotube (SWNT) complexes by adsorption of a blocking reagent. Colloids Surf. B.

[32] Nepal D, Geckeler KE (2006). pH-sensitive dispersion and debundling of single-walled carbon nanotubes: Lysozyme as a tool. Small.

[33] Niidome Y, Wakabayashi R, Goto M, Fujigaya T, Shiraki T (2022). Protein-structure-dependent spectral shifts of near-infrared photoluminescence from locally functionalized single-walled carbon nanotubes based on avidin–biotin interactions. Nanoscale.

[34] Chen C (2013). Recent advances in electrochemical glucose biosensors: A review. RSC Adv..

[35] Zhu Z (2012). A critical review of glucose biosensors based on carbon nanomaterials: Carbon nanotubes and graphene. Sensors.

[36] Holzinger M, Le Goff A, Cosnier S (2014). Nanomaterials for biosensing applications: A review. Front. Chem..

[37] Kurkina T, Vlandas A, Ahmad A, Kern K, Balasubramanian K (2011). Label-free detection of few copies of DNA with carbon nanotube impedance biosensors. Angew. Chem. Int. Ed..

[38] Kurnosov N, Leontiev V, Linnik A, Lytvyn O, Karachevtsev V (2014). Photoluminescence intensity enhancement in SWNT aqueous suspensions due to reducing agent doping: Influence of adsorbed biopolymer. Chem. Phys..

[39] Liu Z, Tabakman S, Welsher K, Dai H (2009). Carbon nanotubes in biology and medicine: In vitro and in vivo detection, imaging and drug delivery. Nano Res..

[40] Mehra NK, Mishra V, Jain N (2014). A review of ligand tethered surface engineered carbon nanotubes. Biomaterials.

[41] Tung NT (2017). Peptide aptamer-modified single-walled carbon nanotube-based transistors for high-performance biosensors. Sci. Rep..

[42] Choudhury D, Biswas S, Roy S, Dattagupta J (2010). Improving thermostability of papain through structure-based protein engineering. Protein Eng. Des. Sel..

[43] Li F-Y, Xing Y-J, Ding X (2007). Immobilization of papain on cotton fabric by sol–gel method. Enzyme Microb. Technol..

[44] Chen Y, Zhou S, Li L, Zhu J-J (2017). Nanomaterials-based sensitive electrochemiluminescence biosensing. Nano Today.

[45] Putzbach W, Ronkainen NJ (2013). Immobilization techniques in the fabrication of nanomaterial-based electrochemical biosensors: A review. Sensors.

[46] Hayashi T, Hirayama C, Iwatsuki M (1992). Papain immobilization onto porous poly (λ-methyl l-glutamate) beads. J. Appl. Polym. Sci..

[47] Kaur G, Bharti S, Tripathi S (2018). Interactions between thioglycolic acid capped CdSe/ZnS nanoparticles and papain. J. Lumin..

[48] Rajalakshmi N, Sundaram P (1995). Stability of native and covalently modified papain. Protein Eng. Des. Sel..

[49] Budama-Kilinc Y (2018). Papain loaded poly (ε-caprolactone) nanoparticles: In-silico and in-vitro studies. J. Fluoresc..

[50] Sheng W, Xi Y, Zhang L, Ye T, Zhao X (2018). Enhanced activity and stability of papain by covalent immobilization on porous magnetic nanoparticles. Int. J. Biol. Macromol..

[51] Sahoo B, Sahu SK, Bhattacharya D, Dhara D, Pramanik P (2013). A novel approach for efficient immobilization and stabilization of papain on magnetic gold nanocomposites. Colloids Surf. B.

