# Supplementary material for: Hybridization of papain molecules and DNA-wrapped single-walled carbon nanotubes evaluated by atomic force microscopy in fluids
Source: Sci Rep. 2023 Mar 24;13:4833. doi: 10.1038/s41598-023-31927-8 (PMC10039081; doi:10.1038/s41598-023-31927-8)
Supplement: Supplementary file 1 — Supplementary Figures. [file 41598_2023_31927_MOESM1_ESM.docx]

**Hybridization of papain molecules and DNA-wrapped single-walled carbon nanotubes evaluated by atomic force microscopy in fluids**

**Masaki Kitamura*, Kazuo Umemura**

Department of Physics, Tokyo University of Science, 1-3 Kagurazaka, Shinjuku, 1628601, Japan

***Corresponding Author.** E-mail: [1222518@ed.tus.ac.jp](mailto:1222518@ed.tus.ac.jp)

**
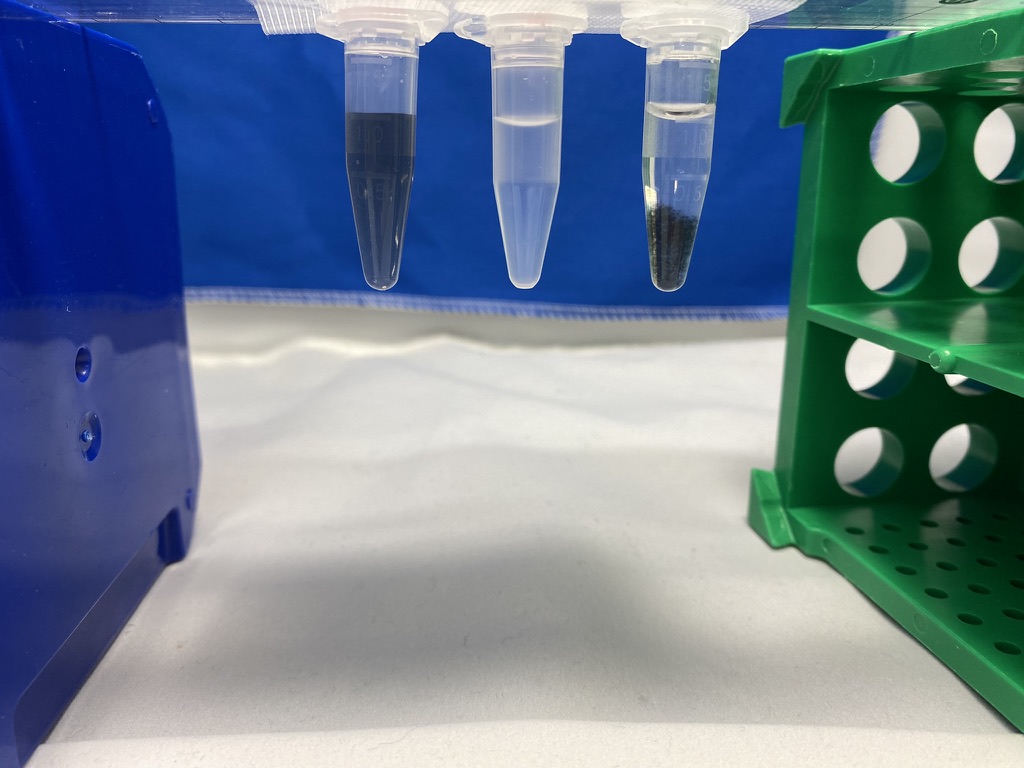
**

Figure S1 The pictures of DNA-SWNT hybrids solution (left), papain solution (middle), and the aggregates of DNA-SWNT hybrids and papain molecules (right).


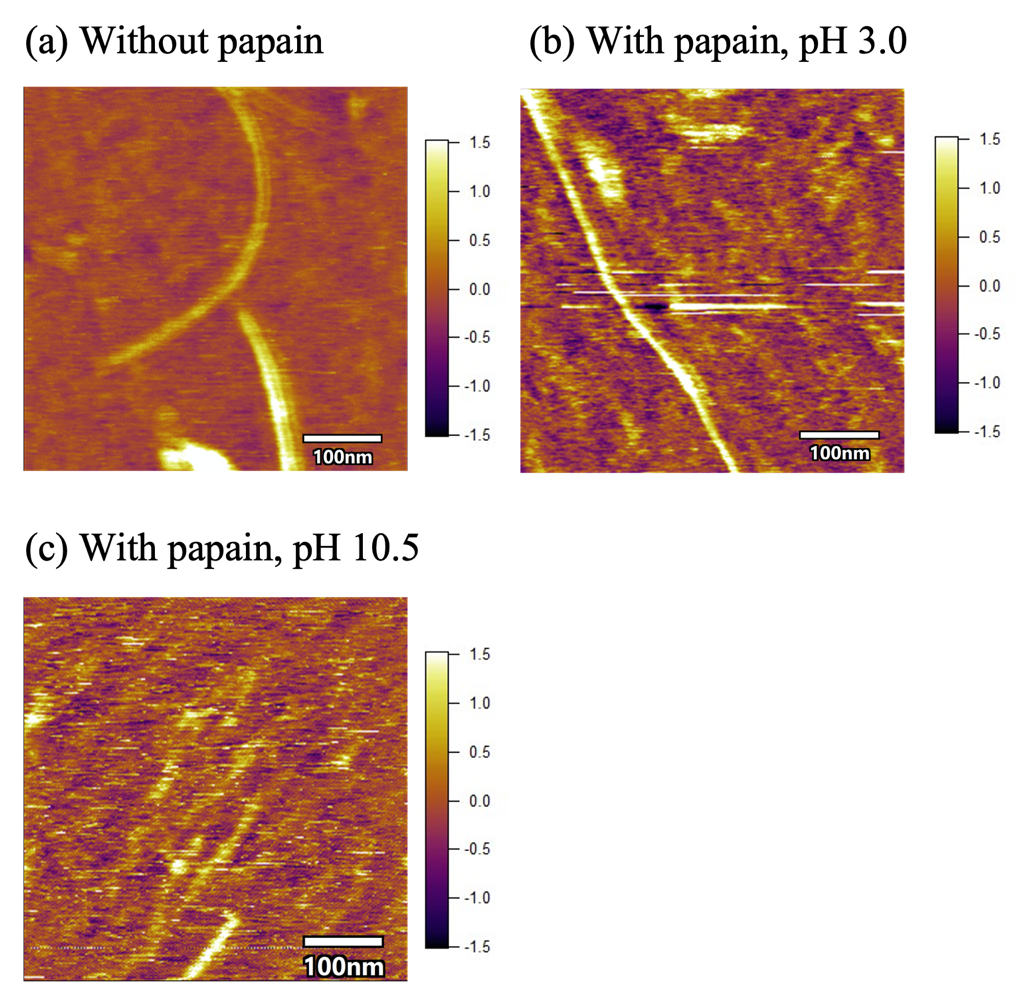


Figure S2 Zoom-ins of AFM images of the DNA-SWNT (a) without papain molecules at pH 3.0 and (b) with papain molecules at pH 3.0 and (c) with papain molecules at pH 10.5.


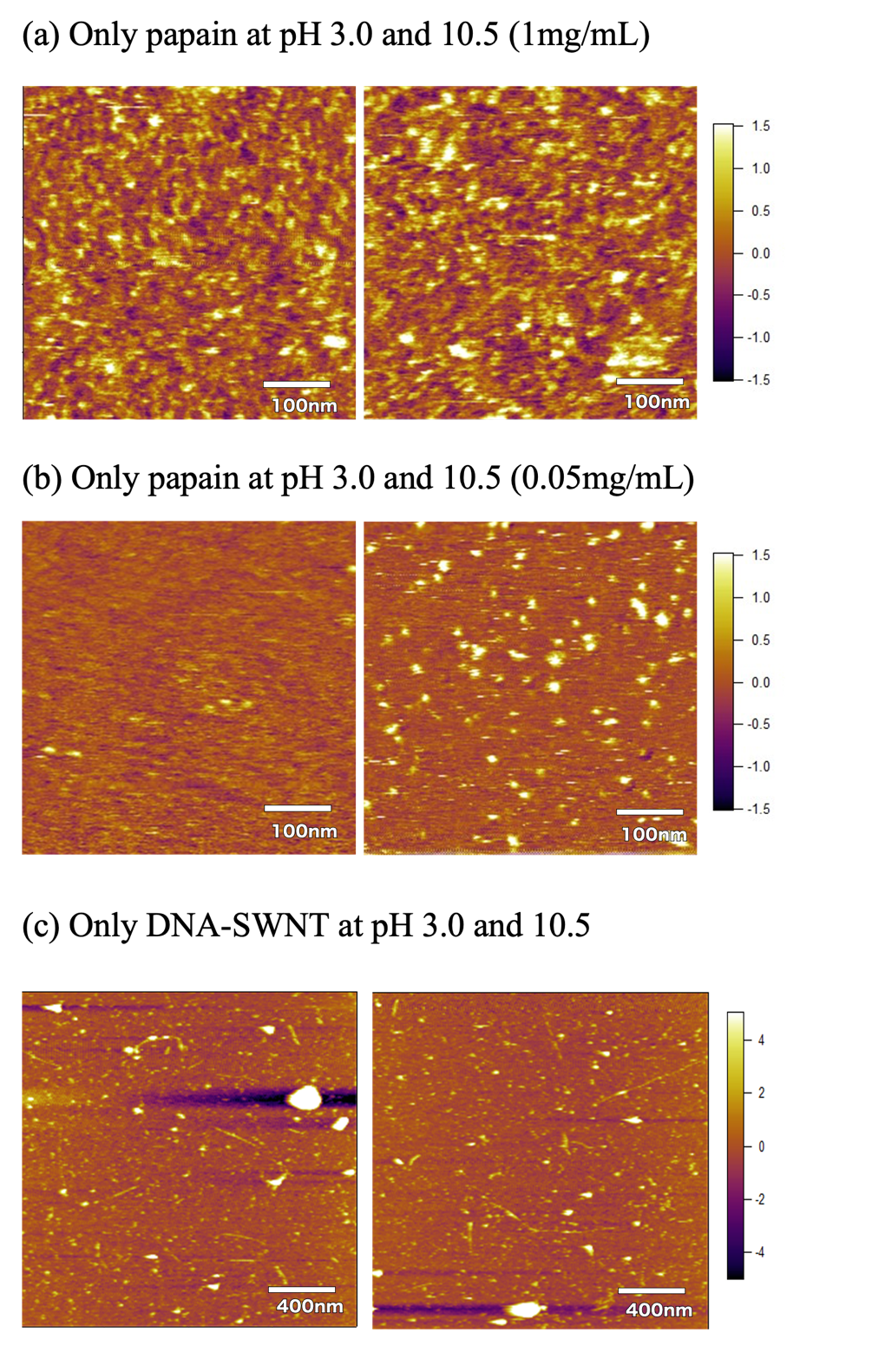


Figure S3 AFM images of the (a) papain molecules at 1.0 mg/mL and (b) papain molecules at 0.05 mg/mL and (c) DNA–SWNTs with papain molecules at pH 3.0 and 10.5. Some dark areas are the artefacts caused by the large structures and these areas are not the natural properties of the mica surface.


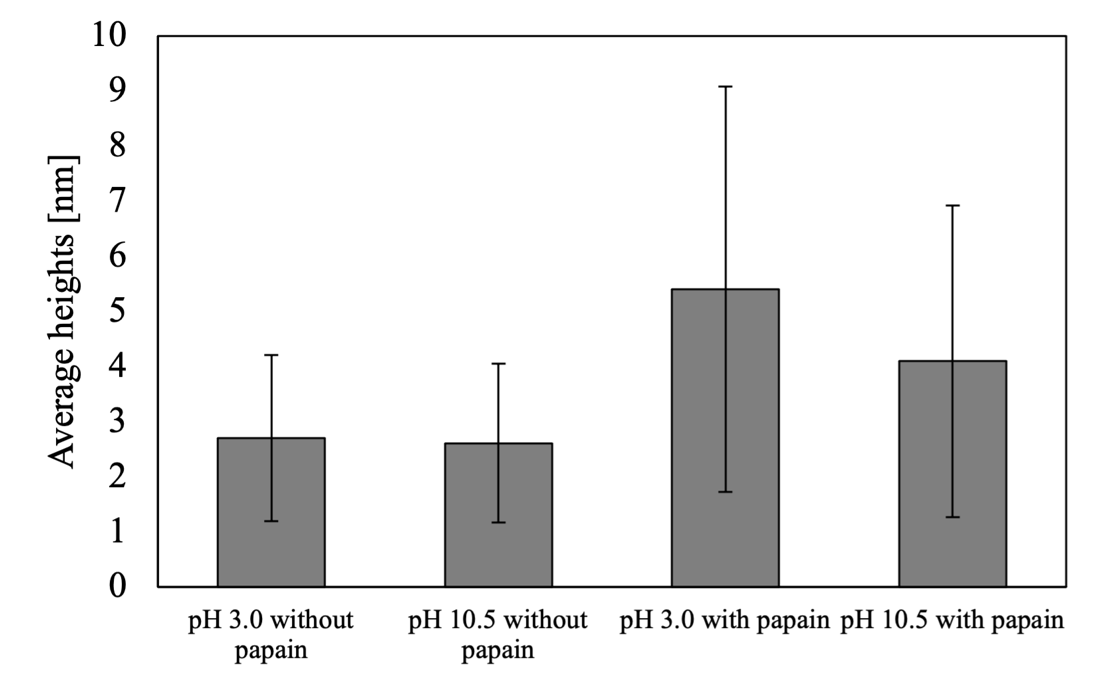


Figure S4 The bar graph of the average heights in scheme 1 with a standard division (the significant differences were confirmed by *t*-tests).


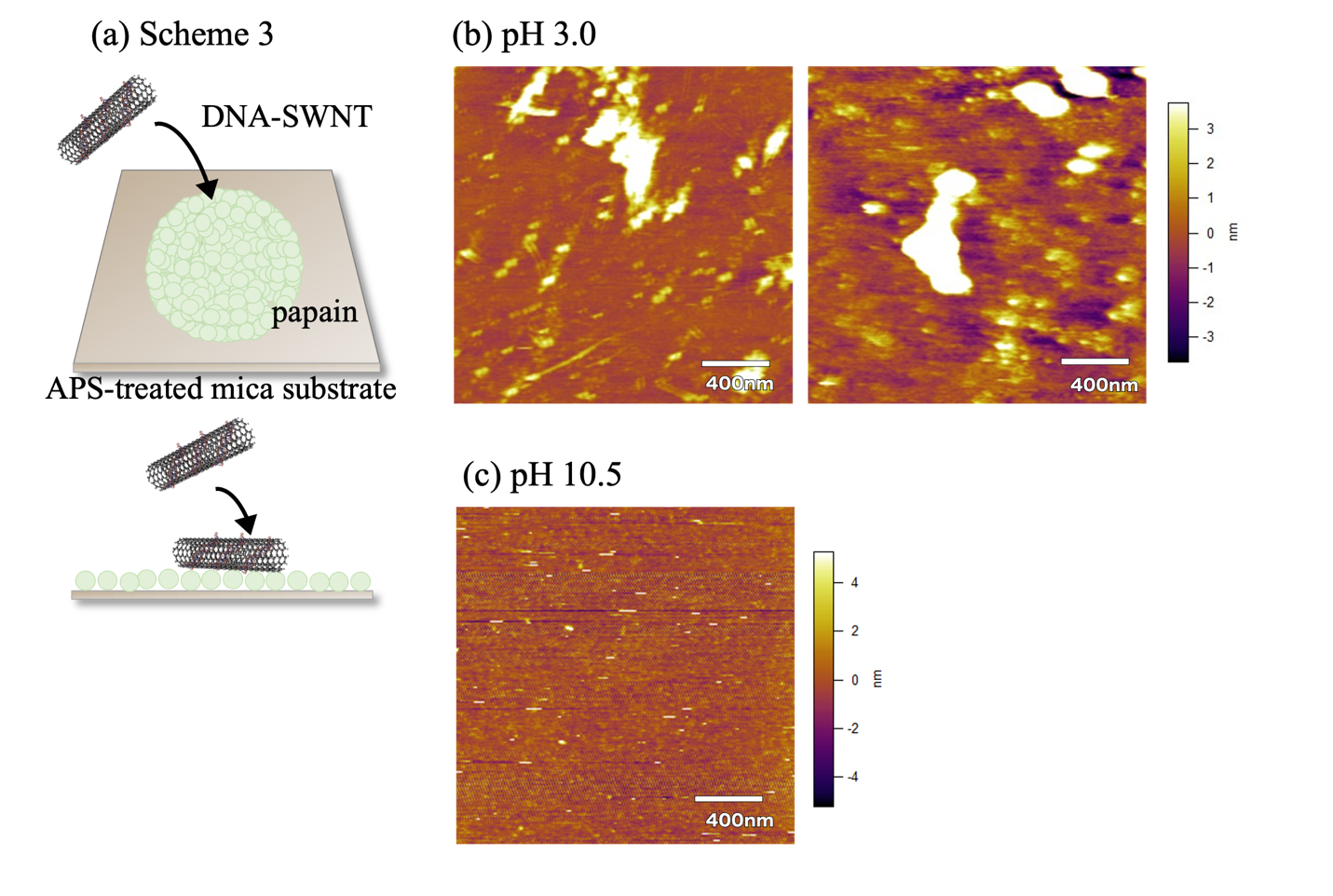


Figure S5. (a) Scheme 3 experiment, where papain molecules were first adsorbed, followed by the injection of DNA–SWNTs. AFM images at (b) pH 3.0 and (c) pH 10.5 after injection.


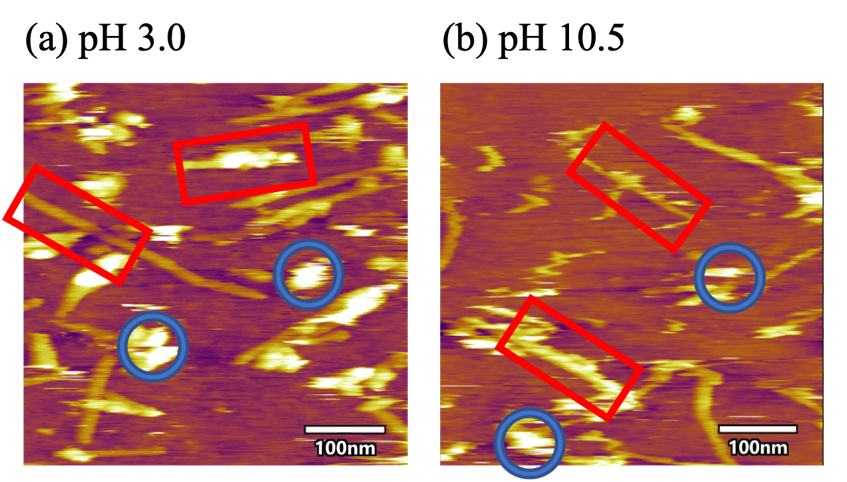


Figure S6 Zoom-ins of AFM images of the DNA-SWNT with papain molecules (a) at pH 3.0 and (b) at pH 10.5.


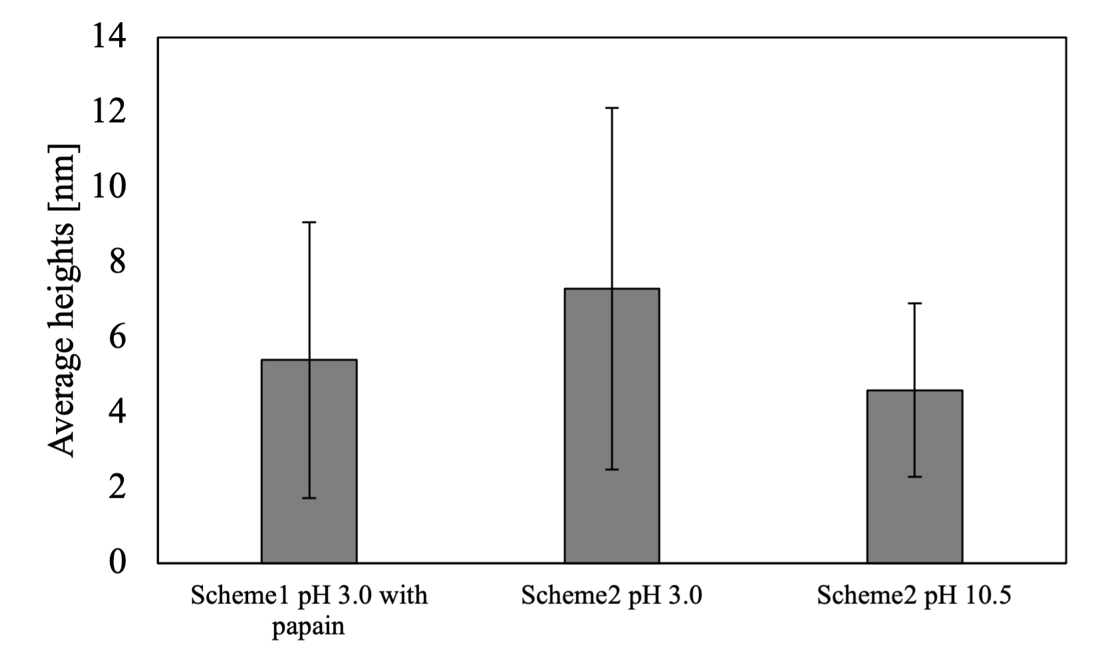


Figure S7 The bar graph of the average heights in scheme 1 and 2 with a standard division (the significant differences were confirmed by *t*-tests).
